# Life expectancy and healthy life expectancy of Japan: the fastest graying society in the world

**DOI:** 10.1186/s13104-016-2281-2

**Published:** 2016-10-28

**Authors:** Shinkan Tokudome, Shuji Hashimoto, Akihiro Igata

**Affiliations:** 1 Department of Nutritional Epidemiology, National Institute of Health and Nutrition, 1-23-1, Toyama, Shinjuku-ku, Tokyo, 162-8636 Japan; 2 Fujita Health University School of Medicine, Toyoake, Japan; 3 Nagoya University of Arts and Sciences, Nisshin, Japan

**Keywords:** Healthy life expectancy, Life expectancy, Non-communicable disease, Quality of death, Quality of life

## Abstract

**Background:**

We appraised time trends of Japanese life expectancy (LE) and healthy life expectancy (HALE) by gender, LE-HALE and (LE-HALE)/LE figures, along with the women–men’s differences.

**Methods:**

Using the Japanese LE and HALE values from 1990 through 2013 by gender in the article by the GBD 2013 DALYs and HALE Collaborators, we examined trends of LE and HALE, and their 5- or 3-year changes. We also probed LE-HALE and (LE-HALE)/LE values, and the women–men’s differences.

**Results:**

LE consistently elongated as reported 76.0, 76.5, 77.6, 78.7, 79.3 and 80.1 years for men from 1990 to 2013; and 82.0, 82.8, 84.3, 85.5, 86.1 and 86.4 years for women, respectively. Both time trends demonstrated a significant linear increase (*p* for trend < 0.001). LE changes were 0.4, 1.1, 1.1, 0.7 and 0.7 years for men, and 0.9, 1.5, 1.2, 0.6 and 0.3 years for women. The trends were statistically significant (*p* < 0.001), except for 2010–2013 partly due to 3-year interval. HALE also steadily lengthened as seen 68.1, 68.4, 69.1, 69.9, 70.8 and 71.1 years for men from 1990 through 2013; and 72.2, 72.9, 74.0, 74.8, 75.4 and 75.6 years for women. Both time trends showed almost a linear increase (*p* < 0.05). HALE changes were 0.4, 0.6, 0.8, 0.9 and 0.3 years for men, and 0.7, 1.0, 0.8, 0.6 and 0.2 years for women, without statistical significant trends. LE-HALE values were 8.0, 8.0, 8.5, 8.8, 8.6 and 8.9 years for men; and 9.7, 9.9, 10.4, 10.7, 10.7 and 10.8 years for women. (LE-HALE)/LE figures were 10.5, 10.5, 10.9, 11.1, 10.8 and 11.2% for men, and 11.9, 12.0, 12.3, 12.5, 12.4 and 12.5% for women. LE women–men’s differences were 5.9, 6.4, 6.8, 6.8, 6.8 and 6.3 years, and the HALE figures were 4.2, 4.5, 4.9, 4.9, 4.6 and 4.5 years.

**Conclusions:**

LE and HALE consistently linearly elongated for both sexes over the study period. Not only LE-HALE but also (LE-HALE)/LE values were still growing for both sexes. Public health measures, nursing-care/services as well as social security schemes are called for to further elevate longevities, HALE in particular, and enhance quality of life and well-being.

## Background

Global burden of disease (GBD) and global, regional, and national trends of life expectancy (LE) (the average number of years expected to live at birth) and healthy life expectancy (HALE) [LE taken into account disability-adjusted life-years (DALYs) at birth, disability-free LE, or health-adjusted LE] [1] in the world from 1990 through 2013 were reported by the GBD 2013 DALYs and HALE Collaborators [2, 3]. They provided valuable information on potential sociodemographic factors pertaining to the longevity figures and their transition, and demonstrated global perspectives for achieving sustainable development goals. In addition, they underscored the need for country- and sociodemographic group-specific assessments of the longevity values.

Approximately 70 years ago (in 1947), Japanese LE was only ca 50 years for men and 54 years for women [4]. In 2013, Japan attained not only the world’s longest LE of 80.1 years for men and 86.4 years for women, but also the highest HALE of 71.1 years for men and 75.6 years for women [2]. LE of >80 years for men was first achieved in Japan. HALE of >70 years was enjoyed by Japanese men along with the men of Singapore (70.8 years) for the first time worldwide.

Thus, it seems informative not only to note trends of LE and HALE by gender, their 5- or 3-year changes [that is, 5 years in length for the first 4 intervals, while 3 years for the last interval (2010–2013)], LE-HALE and (LE-HALE)/LE figures, and their women–men’s differences but also to discuss public health schemes, nursing-care services, and social security measures thus far implemented (or to be launched) for elevating longevities, HALE in particular, and realizing a better quality of life (QOL) and quality of death (QOD).

## Methods

Using the Japanese LE and HALE figures by gender in 1990, 1995, 2000, 2005, 2010 and 2013 in the articles by the GBD 2013 DALYs and HALE Collaborators [2, 3], we probed trends of LE and HALE, along with 5-year changes of 1990–1995, 1995–2000, 2000–2005, 2005–2010 and 3-year change of 2010–2013. We also studied LE-HALE and (LE-HALE)/LE figures, and explored differences in LE and HALE between men and women.

### Statistical analyses

On the basis of linear regression model, each trend of LE and HALE from 1990 through 2013 by gender was examined setting respective calendar years as independent variables in consideration of the survey time interval, and assuming the respective longevity indices weighted by inverse variance as dependent variables.

Using two-sample z-tests, we statistically probed the 5- or 3-year differences of LE and HALE by gender from 1990 through 2013. Assuming LE and HALE are asymptotically distributed according to normal distribution, we estimated the 95% UIs for LE-HALE and (LE-HALE)/LE values along with gender differences in LE and HALE and similarly examined respective differences.

We considered *p* < 0.05 (two-tailed) as statistically significant.

## Results

**Fig. 1:**
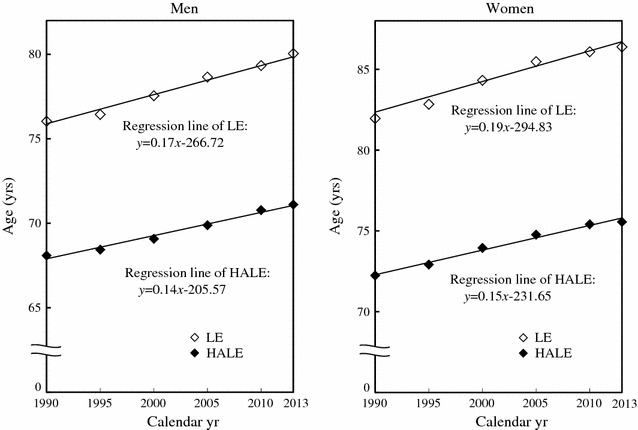
Trends of LE and HALE of Japan from 1990 through 2013

LE consistently elongated as reported 76.04, 76.45, 77.55, 78.66, 79.34 and 80.05 years for men from 1990 through 2013; and 81.96, 82.84, 84.32, 85.48, 86.09 and 86.39 years for women, in that order (Table 1). Both time trends showed a linear increase, and both gradients were statistically significant (*p* for trend < 0.001) (Fig. 1). LE 5-year changes (except for 2010-2013) were 0.4, 1.1, 1.1, 0.7 and 0.7 years for men, and 0.9, 1.5, 1.2, 0.6 and 0.3 years for women, with statistical significance (*p* < 0.001), but not for the last interval due partly to a 3-year difference and wider corresponding 95% UIs.

**Table 1:** Trends and changes of LE and HALE, LE-HALE and (LE-HALE)/LE, and their gender differences in Japan from 1990 through 2013

Longevity index	Calendar year											*p* for trend
	1990	1990–1995	1995	1995–2000	2000	2000–2005	2005	2005–2010	2010	2010–2013	2013	
LE (years)^a,b^												
Men	*76.04*	0.4	*76.45*	1.1	*77.55*	1.1	*78.66*	0.7	*79.34*	0.7	*80.05*	*p* < 0.001
95% UIs^c^	*(75.98–76.10)*		*(76.14–76.57)*		*(77.53–77.58)*		*(78.60–78.71)*		*(79.31–79.36)*		*(79.26–80.84)*	
Women	*81.96*	0.9	*82.84*	1.5	*84.32*	1.2	*85.48*	0.6	*86.09*	0.3	*86.39*	*p* < 0.001
95% UIs	*(81.86–82.05)*		*(82.62–82.94)*		*(84.29–84.35)*		*(85.41–85.54)*		*(86.06–86.11)*		*(85.74–87.12)*	
HALE (years)^a,b^												
Men	*68.09*	0.4	*68.44*	0.6	*69.08*	0.8	*69.89*	0.9	*70.78*	0.3	*71.11*	*p* < 0.05
95% UIs	*(65.83–70.11)*		*(66.07–70.58)*		*(66.67–71.24)*		*(67.31–72.12)*		*(68.20–73.06)*		*(68.50–73.57)*	
Women	*72.24*	0.7	*72.92*	1.0	*73.95*	0.8	*74.77*	0.6	*75.41*	0.2	*75.56*	*p* < 0.05
95% UIs	*(69.38–74.77)*		*(70.05–75.49)*		*(70.94–76.55)*		*(71.66–77.46)*		*(72.34–78.23)*		*(72.46–78.42)*	
LE–HALE (years)												
Men	8.0		8.0		8.5		8.8		8.6		8.9	NS
95% UIs	(5.8–10.1)		(5.7–10.3)		(6.2–10.8)		(6.4–11.2)		(6.1–11.0)		(6.3–11.6)	
Women	9.7		9.9		10.4		10.7		10.7		10.8	NS
95% UIs	(7.0–12.4)		(7.2–12.6)		(7.6–13.2)		(7.8–13.6)		(7.7–13.6)		(7.8–13.9)	
(LE–HALE)/LE (%)												
Men	10.5		10.5		10.9		11.1		10.8		11.2	NS
95% UIs	(7.6–13.3)		(7.5–13.4)		(8.0–13.9)		(8.1–14.2)		(7.7–13.9)		(7.9–14.5)	
Women	11.9		12.0		12.3		12.5		12.4		12.5	NS
95% UIs	(8.6–15.2)		(8.7–15.3)		(9.0–15.6)		(9.1–15.9)		(9.0–15.8)		(9.0–16.1)	
LE (years)												
Women–men	5.9		6.4		6.8		6.8		6.8		6.3	*p* < 0.001
95% UIs	(5.8–6.0)		(6.1–6.7)		(6.7–6.8)		(6.7–6.9)		(6.7–6.8)		(5.3–7.4)	
HALE (years)												
Women–men	4.2		4.5		4.9		4.9		4.6		4.5	NS
95% UIs	(0.7–7.6)		(1.0–8.0)		(1.3–8.5)		(1.1–8.7)		(0.8–8.5)		(0.5–8.4)	

^a^Healthy life expectancy from GBD 2013 DALYs and HALE Collaborators. Global, regional, and national disability-adjusted life years (DALY) for 306 diseases and injuries and healthy life expectancy (HALE) for 188 countries, 1990–2013: quantifying the epidemiological transition. Lancet, Published online August 27, 2015, http://www.dx.doi.org/10.1016/S0140-6736(15)61340-x

^b^Personal communication for the Japanese LE and HALE data of 1995, 2000 and 2010 from Ms. Michelle L. Subart at the Institute for Health Metrics and Evaluation, University of Washington, Seattle, WA, USA (on July 15, 2016)

^c^Uncertainty intervals

HALE also steadily lengthened as seen 68.09, 68.44, 69.08, 69.89, 70.78 and 71.11 years for men from 1990 through 2013; and 72.24, 72.92, 73.95, 74.77, 75.41 and 75.56 years for women. Both time trends manifested almost a linear increase, and both gradients were statistically significant (*p* for trend < 0.05). HALE 5-year changes (except for 2010–2013) were 0.4, 0.6, 0.8, 0.9 and 0.3 years for men, and 0.7, 1.0, 0.8, 0.6 and 0.2 years for women, without statistical significance.

LE-HALE values were 8.0, 8.0, 8.5, 8.8, 8.6 and 8.9 years for men from 1990 through 2013; and 9.7, 9.9, 10.4, 10.7, 10.7 and 10.8 years for women. (LE-HALE)/LE figures were 10.5, 10.5, 10.9, 11.1, 10.8 and 11.2% for men, and 11.9, 12.0, 12.3, 12.5, 12.4 and 12.5% for women. LE women–men’s discrepancies were 5.9, 6.4, 6.8, 6.8, 6.8 and 6.3 years, and the HALE figures were 4.2, 4.5, 4.9, 4.9, 4.6 and 4.5 years.

## Discussion

Although there was an LE drop in 1995, being partly explained by the Great Hanshin-Awaji Earthquake with 6434 victims and three unidentified people [5], LE consistently elongated for both sexes from 1990 through 2013. LE may be determined in part by genetic diatheses, but mostly by modifiable chronic conditions, health behaviors, and socio-economic and environmental factors [6–9]. For further lengthening LE, the following potential factors are to be taken into account. The traditional well-balanced palatable Japanese cuisine has gradually been changing, but retaining higher salt consumption (10.9 g/day on average for men and 9.2 g/day for women in 2014) [10]. Abundant/imbalanced intakes of energy and nutrients (typically affluent/imbalanced consumption of fats and oils, milk and dairy products, and meat instead of fish/shell fish, but lower intake of calcium) seem to be compatible with enhancing trends of metabolic syndrome (obesity, hypertension, dyslipidemia, and insulin resistance). Smoking rates remain higher among industrialized countries (32.2% in men and 8.5% in women), and tobacco-related cancers and diseases are (and will be) frequent. Although the prevalence of daily drinking is declining, only a small fraction of alcohol-dependent patients had received medical care [10, 11]. Sedentary working style, physical inactivity, and greater motorization are prevailing [10, 12]. The participation rates in annual health checkups in the workplace were high, but rather lower in the community, and population-based cancer screening for several sites (such as the stomach, lung, colorectum, and cervix uteri) stay lower. Mental/psychological health disorders (including depression) are big issues in the workplace due to overtime, stressful working environment and relationship dynamics within the workplace [12, 13].

HALE also manifested an upward increase for both sexes over the study period. Neither *p* for trend nor the difference of regression coefficient between LE and HALE was significant, but LE-HALE and (LE-HALE)/LE values were steadily gaining, as also reported by Salomon and colleagues that “HALE increased more slowly than did LE [14].” Also of note is that LE-HALE and (LE-HALE)/LE in women were greater than in men, which should be taken into account as they are directly involved in elderly women’s QOL. In turn, Japan ranks high overall, ranking 8th, on the Global AgeWatch Index 2015 [15]. Favorable scores have been attained in two domains (Health domain [including longevities] and Capability domain), but have stayed behind in two domains (Income domain and Enabling societies and environment domain), manifesting *age* and *gender* inequities in working opportunity and income, coverage of pension and welfare, and hidden poverty [16]. Namely, HALE may be brought about by not only health-care and rehabilitation but also social security schemes, such as the Pension System, Long-term Nursing-Care, Health and Welfare Services, and Public Assistance [17]. Multi-phasic comprehensive interventions are called for to reduce years lived with disability (YLDs) (i.e., elongate HALE, and compress morbidity or LE-HALE), because people should spend 8.9 YLDs (for men) and 10.8 YLDs (for women) (being more than 10% of the period of his/her life) in poor health.

The proportion of the elderly aged 65 years or over was ca 25% in 2013, against 60% for working population aged 15–64 years [18]. The expenditure of benefits for the elderly group is steeply rising, and the burdens of work force and municipal/governmental finance are concurrently growing. Actually, the national health-care expenditure was Jpn Yen 40 trillion [ca US $333 billion (under 1$ = Jpn Yen 120)] in 2014, accounting for more than 40% of the national budget [19]. Japan is facing “the Year 2025 Problem,” when two people in its work force should be able to support one elderly person, and the expenditure will be Jpn Yen 50 trillion (half of the national budget). By this year, as a typical delimiting of time, the baby-boomers will join the old group. Thanks to “low fertility and low mortality,” it is forecast that the proportion of the elderly will be steadily growing, while that of the work force is tapering off, and the ratio is going to increase at least until 2060 [18]. The overall situation appears to be worsening as Japan is clearly moving towards an unheard-of super-aging crisis: too fast for Japanese society to catch-up with. In addition to innovative “core” structural reforms of the national administration and finance, reconstructions of social security plans must be executed, such as deferring of the retirement age, postponing the age for receiving pensions, advanced enrollment of “minorities” (the elderly and women), working on a regular basis, “equal labor, equal pay” for full-time and part-time workers, and effective/efficient use of Public Assistance for the needy elderly [20–22].

Japanese Universal Health Coverage (UHC) (Health Insurance for All, Nationwide Health Insurance Scheme) has largely contributed to enhance HALE as well as LE [20, 21]. The UHC may be a global model of health-care for its low-cost with equity. However, continuing structural reforms of the UHC and health-care service systems have been made for the reduction of health-care costs, withholding of the elderly’s health-care benefits, and so forth. Shared risk communication and securing a second opinion seem essential, but it is troublesome to realize that the UHC allows free and/or redundant (at times useless/unnecessary) visits to medical clinics/facilities, and repeated medical examinations and medicines [23]. The family/general physician system is developing, but the Medical Care Zone Scheme is not necessarily working well [21, 24]. Using the Social Security and Tax Number System (My Number System) just enacted [25], the reduction of health-care costs could partly be achieved by record linkage with electronic health records to be stored in a unique IC card (or in cloud computing). Needless to say, this system must be launched under a national consensus, and should be handled under strict security and confidentiality.

QOL now includes several aspects; not only quality of biological life and quality of daily life but also quality of existence are of importance to fulfill one’s life, to live and die with dignity, and to finally realize better QOD. Recently, the Economist Intelligent Unit of the UK Economist magazine ranked Japan’s QOD index 2015 as 14th in the world [26]. The profiles of Japanese Affordability of care, Quality of care, and Community engagement were satisfactory, while those of Human resources and Palliative and health-care environment were rather unsatisfactory, so the ameliorations are urgent. Much remains to be improved in terms of QOD as well as HALE and QOL from the aspect of the well-being index of the elderly.

## Conclusions

Japan has achieved the world’s longest HALE as well as LE for both sexes in 2013. The increasing trends from 1990 through 2013, and significant changes were still being demonstrated. Not only long-term health-care strategies but also comprehensive community health promotion and social security services should be undertaken to further reduce mortality gap/health gap, elevate HALE, and enhance QOL and QOD in Japan.

## Abbreviations

DALYs: disability-adjusted life years
GBD: global burden of disease
HALE: healthy life expectancy
LE: life expectancy
MHLW: Ministry of Health, Labour and Welfare
QOD: quality of death
QOL: quality of life
UHC: universal health coverage
UI: uncertainty interval
YLDs: years lived with disability
